# Promising microRNAs in pre-diagnostic serum associated with lung cancer up to eight years before diagnosis: a HUNT study

**DOI:** 10.1007/s00432-024-05882-4

**Published:** 2024-07-20

**Authors:** Ioannis Fotopoulos, Olav Toai Duc Nguyen, Therese Haugdahl Nøst, Maria Markaki, Vincenzo Lagani, Robin Mjelle, Torkjel Manning Sandanger, Pål Sætrom, Ioannis Tsamardinos, Oluf Dimitri Røe

**Affiliations:** 1 Department of Computer Science, University of Crete, 700 13 Heraklion, Crete Greece; 2 Department of Clinical and Molecular Medicine, NTNU-Norwegian University of Science and Technology, Prinsesse Kristinas Gate 1, 7030 Trondheim, Norway; 3 Cancer Clinic, Levanger Hospital, Nord-Trøndelag Health Trust, Kirkegata 2, 7600 Levanger, Norway; 4 Department of Community Medicine, Faculty of Health Sciences, UiT The Arctic University of Norway, P.O. Box 6050 Langnes, 9037 Tromsø, Norway; 5 K.G. Jebsen Center for Genetic Epidemiology, Department of Public Health and Nursing, NTNU-Norwegian University of Science and Technology, Postboks 8905, 7491 Trondheim, Norway; 6 Institute of Applied and Computational Mathematics, FORTH, 700 13 Heraklion, Crete Greece; 7 Institute of Chemical Biology, Ilia State University, 3/5, Kakuca Cholokashvili Ave, Tbilisi, Georgia; 8 Biological and Environmental Sciences and Engineering Division (BESE), King Abdullah University of Science and Technology KAUST, Thuwal, Saudi Arabia; 9 SDAIA-KAUST Center of Excellence in Data Science and Artificial Intelligence, 23952 Thuwal, Saudi Arabia; 10 Bioinformatics Core Facility, NTNU-Norwegian University of Science and Technology, 7491 Trondheim, Norway; 11 Department of Computer Science, Norwegian University of Science and Technology, 7491 Trondheim, Norway; 12 JADBio Gnosis DA S.A., STEP-C, N. Plastira 100, 700-13 Heraklion, Greece; 13 Department of Oncology and Clinical Cancer Research Center, Aalborg University Hospital, 9000 Aalborg, Denmark; 14 Department of Clinical Medicine, Aalborg University Hospital, 9000 Aalborg, Denmark

**Keywords:** Serum early detection, Risk model for lung cancer, MicroRNA sequencing

## Abstract

**Introduction:**

Blood biomarkers for early detection of lung cancer (LC) are in demand. There are few studies of the full microRNome in serum of asymptomatic subjects that later develop LC. Here we searched for novel microRNA biomarkers in blood from non-cancer, ever-smokers populations up to eight years before diagnosis.

**Methods:**

Serum samples from 98,737 subjects from two prospective population studies, HUNT2 and HUNT3, were considered initially. Inclusion criteria for cases were: ever-smokers; no known cancer at study entrance; 0–8 years from blood sampling to LC diagnosis. Each future LC case had one control matched to sex, age at study entrance, pack-years, smoking cessation time, and similar HUNT Lung Cancer Model risk score. A total of 240 and 72 serum samples were included in the discovery (HUNT2) and validation (HUNT3) datasets, respectively, and analysed by next-generation sequencing. The validated serum microRNAs were also tested in two pre-diagnostic plasma datasets from the prospective population studies NOWAC (n = 266) and NSHDS (n = 258). A new model adding clinical variables was also developed and validated.

**Results:**

Fifteen unique microRNAs were discovered and validated in the pre-diagnostic serum datasets when all cases were contrasted against all controls, all with AUC > 0.60. In combination as a 15-microRNAs signature, the AUC reached 0.708 (discovery) and 0.703 (validation). A non-small cell lung cancer signature of six microRNAs showed AUC 0.777 (discovery) and 0.806 (validation). Combined with clinical variables of the HUNT Lung Cancer Model (age, gender, pack-years, daily cough parts of the year, hours of indoor smoke exposure, quit time in years, number of cigarettes daily, body mass index (BMI)) the AUC reached 0.790 (discovery) and 0.833 (validation). These results could not be validated in the plasma samples.

**Conclusion:**

There were a few significantly differential expressed microRNAs in serum up to eight years before diagnosis. These promising microRNAs alone, in concert, or combined with clinical variables have the potential to serve as early diagnostic LC biomarkers. Plasma is not suitable for this analysis. Further validation in larger prospective serum datasets is needed.

**Supplementary Information:**

The online version contains supplementary material available at 10.1007/s00432-024-05882-4.

## Introduction

Early diagnosis is a prerequisite for curative treatment of lung cancer. However, whilst Stage I five-year survival is more than 80%, the overall survival of lung cancer is as low as 20%, indicating that the majority are detected too late (National Cancer Institute 2022).

MicroRNAs are small non-coding RNAs with multiple and important functions in the body, including cancer development (Peng and Croce 2016). They are also differentially expressed in lung cancer tumors versus normal tissues and several studies have found circulating microRNA candidates for various types of cancer (Condrat et al. 2020; Kim and Croce 2023). MicroRNAs in serum tend to be stable over time when frozen and therefore can be useful as circulating biomarkers (Matias-Garcia et al. 2020). Currently, only a few types of tests have been validated in pre-diagnostic blood samples for lung cancer risk evaluation or diagnosis, but none are widely used in the clinic (Montani et al. 2015; Seijo et al. 2019; Sozzi et al. 2014). There are several reasons why this may have been a problem, including lack of pre-diagnostic samples, different analytical techniques, quality of the samples and quality of the clinical data. Moreover, lung cancer is a very heterogeneous disease, where more and more molecular subtypes are discovered. There are also issues regarding confounding microRNA signatures due to different smoking profiles between cases and controls, controls may be never-smokers, that may affect those profiles profoundly. Moreover, there is also a lack of subtype-specific profiling in circulating microRNAs in future lung cancer patients. Currently there are several microRNA candidates from various groups that are non-overlapping and not validated (Bottani et al. 2019; Sozzi et al. 2014; Ying et al. 2020).

Here we present a prospective, matched case–control study on adenocarcinoma (AD), squamous cell carcinoma (SQ) and small-cell lung cancer (SCLC) genome-wide microRNA sequencing of pre-diagnostic serum samples up to 8 years before diagnosis. MicroRNAs of interest were validated in three independent datasets, one dataset with pre-diagnostic serum samples up to 3.8 before diagnosis and two datasets from other population-based studies with pre-diagnostic plasma samples up to 5 years before diagnosis. Furthermore, we evaluated the lung cancer predictiveness of these validated microRNAs by combining them with or without the original eight clinical variables of the HUNT Lung Cancer Model (sex, age, body-mass index (BMI), pack-years, number of cigarettes per day, quit time in years, hours of daily indoors smoke exposure and history of daily cough in periods through the year) (Markaki et al. 2018).

## Methods

Two independent serum sample sets were selected from the HUNT2 (discovery) and HUNT3 (validation) population studies, respectively, and stored at − 80 ℃. The selection, RNA-extraction, preparation of the samples and the next generation sequencing were performed as two separate experiments at two different time points using next-generation sequencing technology. A validation analysis was also performed in two pre-diagnostic plasma sample sets from the prospective population studies NOWAC and NSHDS.

### Discovery dataset in pre-diagnostic serum

The discovery dataset was extracted from the HUNT2 study, a prospective, well curated population study in Norway, including data from questionnaires, interviews, clinical measurements and a serum biobank of all these individuals. The HUNT2 enrolled and examined 65,237 people aged > 20 years in 1995–1997 and followed up until 31.12.2011 (Krokstad et al. 2013). This biobank was linked to the National Cancer Registry by the unique personal identification number. After a median follow-up of 15.2 years, 583 lung cancer cases had been diagnosed in this population, and 552 (94.7%) of these were current or former smokers (ever-smokers). Inclusion criteria were the following: no active cancer at inclusion, former or current smokers, diagnosed with lung cancer less than eight years after serum sampling; available clinical variables as age, sex, pack-years, smoking quit time and body-mass index (BMI). The histological subtype should be specified, and the groups should have an equal number of cases and matched controls. In the discovery dataset in total 240 individuals were selected, all 120 cases histology verified: 40 AD, 40 SQ and 40 SCLC (ICD7 code 1621). Consequently, the non-small cell lung cancer (NSCLC) cases consisted of the combination of the AD plus SQ, 80 cases and 80 matched controls. The matched controls were individuals that did not develop lung cancer or any other cancer in the follow-up period, matched on age at participation in the HUNT2 study ± 2 years, pack years ± 2, quit time ± 2 years, sex and HUNT Lung Cancer Model risk score (Markaki et al. 2018). The R package Hmisc (https://cran.r-project.org/web/packages/Hmisc/index.html) was used to identify the controls.

Due to lack of standard staging information, the information provided from the Norwegian Cancer Registry was used (Supplementary Table 1). The Code 0–1 was used as a surrogate marker for Stage I-IIB (termed “non-metastatic”) and Code 2–4 as Stage III-IV (“metastatic”) (Supplementary Table 1). Some cases did not have staging information. Clinically relevant contrasts such as cases versus controls or histological or stage subgroups (metastatic and non-metastatic) versus controls were analysed. The groups were contrasted to their respective matched controls but also to larger groups of controls, including all controls (e.g., AD versus AD controls, AD versus all controls, ADnon-metastatic vs ADnon-metastatic controls, ADnon-metastatic vs AD controls, ADnon-metastatic vs all controls) (Supplementary File 1 and 2). Univariate analysis was also performed in males versus females and current versus former smokers for both cases and control groups, respectively (Supplementary File 2).

### Validation in pre-diagnostic serum samples

There were no overlapping subjects in the HUNT discovery and validation datasets. Validation serum dataset was extracted from the prospective HUNT3 study, where the total county population age > 20 years was invited to participate. Clinical data and serum were collected 2005–2008 from 33,500 unique participants and followed up until 31.12.2011 (Krokstad et al. 2013; Technology NUoSa 2020). Due to the data cutoff time we had only cases with less than six years to diagnosis. Among these participants, we identified 12 with AD, 12 SQ and 12 SCLC. For each subject a non-cancer control that did not develop any type of cancer within six years was identified, matched for age, sex, smoking (pack-years) quit time and HUNT Lung Cancer Model risk score (Markaki et al. 2018).

### Validation in pre-diagnostic plasma samples

The candidate serum microRNAs were also tested in two pre-diagnostic plasma datasets collected from participants in the prospective population studies NOWAC, Norway (n = 266, all women), and NSHDS, Västerbotten County, Sweden (n = 258, both men and women). In both studies, plasma samples were collected from participants in 2003–2006 in NOWAC and 1988–2016 in NSHDS. Lung cancer cases were identified using linkages to national cancer registries and one matched control was identified for each case within the respective study. The interval from blood sampling to time to diagnosis was less than five years for both studies and all histological subtypes were represented in the sample. Smoking status was not matched for and there were 11% and 43% never smokers among cases and controls, respectively, in NOWAC and the corresponding numbers in NSHDS were 11% and 37%. The never smokers were excluded in this validation, only the results for the 28% and 26% former smokers among cases and controls and 62% and 31% current smokers among cases and controls in NOWAC, and the 34% and 38% former smokers and 54% and 25% current smokers correspondingly in NSHDS, were included in these analyses. For further details, see Nøst et al (2023).

### microRNA analysis

Isolation of RNA from 200 μl serum and next generation sequencing was performed according to Mjelle et al. (2017). Small RNA sequencing data were processed according to Mjelle et al. (2017). to generate expression matrices for mature microRNAs. The microRNA analyses in the NOWAC and NSHDS followed the same protocols, and the sequencing experiments were performed in the same laboratory as those for the HUNT samples. For further details, see Nøst et al (2023).

### Statistical analysis

The association of each microRNA with each clinical outcome was assessed using the moderated t-statistics implemented in the R package limma (Ritchie et al. 2015). A variance-stabilization transformation was applied on the microRNA expression values by modelling the mean–variance relationship of the log-counts (Law et al. 2014; Liu et al. 2015) (see Supplementary). The microRNAs analysed were filtered with reads > 0 and > 1 in the minimum sample size of the lung cancer subtype groups as described here (Law et al. 2018), and all p values were adjusted for multiple testing with the Benjamini–Hochberg method (Benjamini and Hochberg 1995).

All univariate analyses were further adjusted for smoking status (current versus former), sex, age at blood sampling, and RNA library size (see Supplementary). To be considered as validated, findings needed to be identified as statistically significant in both the exploration and validation dataset. Association with false discovery rate (FDR) < 0.25 was deemed statistically significant (see Supplementary). The significant validated microRNAs with mean raw microRNA count > 0 and area under the ROC curve (AUC) > 0.60 within each contrast in the discovery dataset, were included as candidate biomarkers. The same analysis was applied in the validation cohort, and microRNA expressed in the same direction as in the discovery dataset were selected. Kaplan–Meier curves were assessed with the log-rank test (Mantel 1966). For the validation in the plasma samples the following model was estimated: miRexp ~ groups + AgeScaled + Sex + L1 + SmokingStatus + scale(log2(libSize)). Here, the groups variable represents case/control status; AgeScaled, the age at diagnosis normalized by the R-function scale; Sex, the participant's sex; L1 the study (NOWAC or NSHDS); SmokingStatus as binary smoking status (either current or former); and libSize is the total miRNA count per sample. P value < 0.05 was considered statistically significant. The R Statistical Software version 4.2.1 (2022-06-23) was used for all analyses (Team 2018).

The shrinkage coefficient method applied to logistic regression as described previously (Markaki et al. 2018), was used to develop multivariable predictive models of the validated differentially expressed microRNA alone or in combination of the original eight clinical variables in the HUNT Lung Cancer Model (Markaki et al. 2018). An evaluation of the lung cancer predictive performance of the multivariable models was performed on the complete discovery and validation datasets.

## Results

The two serum datasets were well balanced with no significant differences regarding age at entrance into the study, smoking behaviour, indoor smoke exposure, cough history, body mass index (BMI) and HUNT Lung Cancer risk score between cases and controls (Table 1).

One AD sample in the discovery dataset was not successfully processed and removed from further analysis with its matched control. There were 119 and 36 matched pairs in each dataset, respectively (Table 1).

**Table 1:** Clinical and demographic characteristics of cases and controls in the discovery and validation serum dataset

	Discovery dataset			Validation dataset		
	Controls n = 119	Cases n = 119	p value	Controls n = 36	Cases n = 36	p value
Gender			1*			1*
Male	77	77		20	20	
Female	42	42		16	16	
Median age, year (range)	63.9 (34.6–86.2)	63.9 (34.6–87.6)	0.99**	68.3 (53.9–82.3)	67.7 (53.6–81.5)	0.99**
Pack-years	33.8 (0.9–107.6)	25.0 (0.5–84.0)	0.986**	27.95 (7.94–75.6)	25.85 (10.36–75.0)	0.884**
Smoking history			1*			1*
Current smoker	88	87		25	24	
Former smoker	31	32		11	12	
Smoking cessation, year (range)	0.0 (0.0–40.0)	0.0 (0.0–40.0)	0.99**	0.0 (0.0–44.5)	0.0 (0.0–30.2)	0.98**
Cough daily			0.244*			NA
Yes	28	36		NA	NA	
No	90	82		NA	NA	
Body mass index (range)	25.7 (18.4–37.7)	25.5 (18.5–41.3)	0.887**	25.95 (15.3–35.7)	25.40 (19.4–36.4)	0.982**
HUNT risk calculator score (range)	0.0125 (0.0003–0.105)	0.0160 (0.00025–0.127)	0.795**	NA	NA	NA
Lung cancer subtypes						
Adenocarcinoma (AD)	NA	39	NA	NA	12	NA
Squamous cell carcinoma (SQ)	NA	40		NA	12	
Small cell lung cancer (SCLC)	NA	40		NA	12	

The variables in the table except the subtypes are included in the HUNT Lung Cancer Model risk score. The percent lung cancer risk in 6 years is the HUNT risk calculator score × 100 (e.g. risk score 0.0125 = 1.25% risk)

*NA* not applicable due to lack of variables

*Chi square statistical test

**KS statistical test

The stage distribution among the three subtypes in the discovery dataset was > 50% metastatic in the AD and SCLC and only 28% in the SQ. The validation dataset showed less metastatic AD, 33% versus 59% and more metastatic SQ 42% versus 28% (Supplementary Table 3).

Time to diagnosis from serum sampling in cases varied between subtypes and datasets, where the median time to diagnosis was 5.12 years (mean = 4.56, range 0.08–8.19) in the discovery dataset and 1.70 years (mean = 1.58, range 0.13–3.76) in the validation dataset (Supplementary Fig. 2).

### RNA expression

The ranking of the 100 highest expressed microRNAs in the discovery dataset showed a similar downward trend as the validation serum dataset (Supplementary Fig. 3). The ranking of expression levels of the top five microRNAs was identical between the two datasets, except one (Supplementary Fig. 4). The number of expressed microRNAs that were kept for the analysis according to the filtering criteria were 1200 out of 1615 and 462 out of 566, for the discovery and validation datasets, respectively.

### Discovery dataset univariate analysis

The univariate analysis in the 37 lung cancer related contrasts showed 480 statistically significant differentially expressed (SDE) microRNAs found in 37 different lung cancer associated contrasts at 0.25 FDR level (Supplementary Excel file 2).

### Pre-diagnostic serum validation dataset in HUNT3

**Fig. 1:**
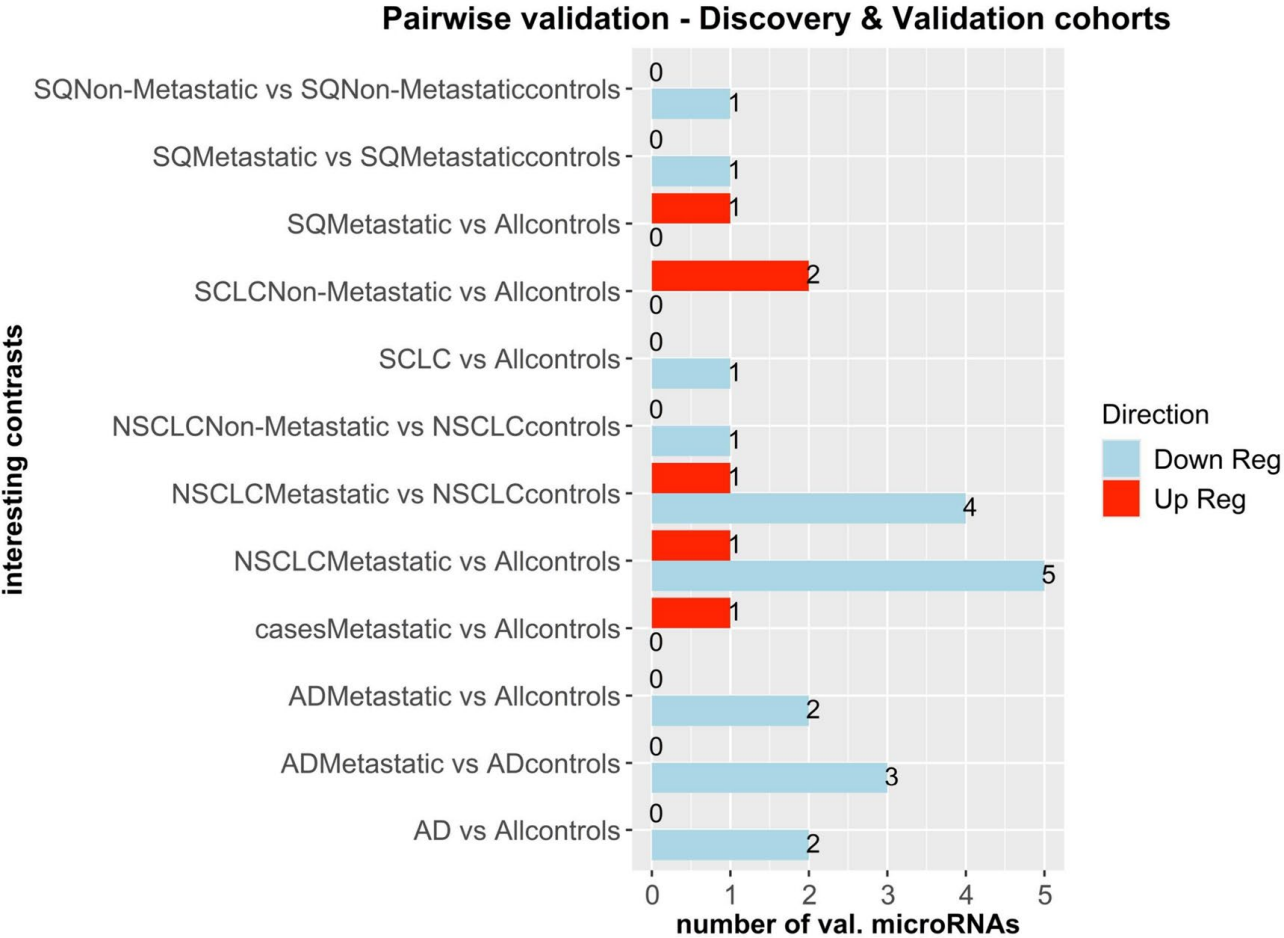
Common SDE microRNAs in the discovery and validation dataset with AUC > 0.6 in both

The multivariate and cross validation analysis of SDE microRNAs in the discovery and validation pre-diagnostic serum datasets, yielded 15 microRNAs in 12 unique contrasts (Fig. 1 and Table 2) encompassing all the main histological lung cancer subtypes. All microRNA in both discovery and validation datasets had AUC > 0.6, except one (Table 2). Four microRNAs were upregulated and 11 were downregulated.

**Table 2:** A total of 15 unique single significantly differentially expressed (SDE) microRNAs in 12 unique contrasts in the discovery and the validation serum datasets. N = cases vs. controls

Contrasts	SDE.miRNAs	Up/down regulated	Discovery serum dataset				Validation serum dataset			
			N	AUC	p value	Adusted. p value	N	AUC	p value	Adjusted p value
AD vs Allcontrols	miR-1228-5p	Down	39 vs 119	0.610	0.009	0.187	12 vs 36	0.604	0.039	0.149
AD vs Allcontrols	miR-191-3p	Down	39 vs 119	0.647	0.000	0.003	12 vs 36	0.648	0.037	0.149
ADMetastatic vs ADcontrols	miR-191-3p	Down	23 vs 39	0.611	0.030	0.239	4 vs 12	0.854	0.002	0.037
ADMetastatic vs ADcontrols	miR-20a-5p	Down	23 vs 39	0.637	0.002	0.053	4 vs 12	0.771	0.008	0.061
ADMetastatic vs ADcontrols	miR-511-5p	Down	23 vs 39	0.624	0.020	0.196	4 vs 12	0.854	0.002	0.037
ADMetastatic vs Allcontrols	miR-191-3p	Down	23 vs 119	0.659	0.003	0.115	4 vs 36	0.826	0.003	0.030
ADMetastatic vs Allcontrols	miR-20a-5p	Down	23 vs 119	0.634	0.003	0.115	4 vs 36	0.736	0.012	0.056
casesMetastatic vs Allcontrols	miR-1306-5p	Up	55 vs 119	0.621	0.015	0.219	17 vs 36	0.691	0.003	0.013
NSCLCMetastatic vs Allcontrols	miR-103a-3p	Down	34 vs 119	0.672	0.014	0.204	9 vs 36	0.713	0.070	0.192
NSCLCMetastatic vs Allcontrols	miR-1306-5p	Up	34 vs 119	0.629	0.011	0.185	9 vs 36	0.704	0.043	0.150
NSCLCMetastatic vs Allcontrols	miR-185-5p	Down	34 vs 119	0.663	0.004	0.119	9 vs 36	0.898	0.016	0.088
NSCLCMetastatic vs Allcontrols	miR-191-3p	Down	34 vs 119	0.636	0.001	0.063	9 vs 36	0.744	0.001	0.014
NSCLCMetastatic vs Allcontrols	miR-191-5p	Down	34 vs 119	0.609	0.018	0.223	9 vs 36	0.614	0.082	0.192
NSCLCMetastatic vs Allcontrols	miR-20a-5p	Down	34 vs 119	0.621	0.003	0.101	9 vs 36	0.608	0.090	0.194
NSCLCMetastatic vs NSCLCcontrols	miR-103a-3p	Down	34 vs 79	0.662	0.011	0.132	9 vs 24	0.690	0.103	0.225
NSCLCMetastatic vs NSCLCcontrols	miR-1306-5p	Up	34 vs 79	0.626	0.012	0.136	9 vs 24	0.648	0.117	0.225
NSCLCMetastatic vs NSCLCcontrols	miR-185-5p	Down	34 vs 79	0.646	0.005	0.086	9 vs 24	0.907	0.028	0.154
NSCLCMetastatic vs NSCLCcontrols	miR-191-3p	Down	34 vs 79	0.614	0.010	0.124	9 vs 24	0.769	0.001	0.027
NSCLCMetastatic vs NSCLCcontrols	miR-20a-5p	Down	34 vs 79	0.642	0.001	0.035	9 vs 24	0.648	0.031	0.154
NSCLCNon-Metastatic vs NSCLCcontrols	miR-144-5p	Down	34 vs 79	0.624	0.025	0.213	10 vs 24	0.796	0.012	0.250
SCLC vs Allcontrols	miR-487a-3p	Down	40 vs 119	0.682	0.000	0.039	12 vs 36	0.602	0.035	0.069
SCLCNon-Metastatic vs Allcontrols	miR-152-3p	Up	10 vs 119	0.682	0.003	0.071	4 vs 36	0.715	0.015	0.173
SCLCNon-Metastatic vs Allcontrols	miR-598-3p	Up	10 vs 119	0.631	0.011	0.177	4 vs 36	0.729	0.017	0.173
SQMetastatic vs Allcontrols	miR-4485-3p	Up	11 vs 119	0.788	0.002	0.225	5 vs 36	0.500	0.072	0.072
SQMetastatic vs SQMetastaticcontrols	miR-616-3p	Down	11 vs 11	0.620	0.025	0.208	5 vs 5	1,000	0.016	0.199
SQNon-Metastatic vs SQNon-Metastaticcontrols	miR-8072	Down	24 vs 24	0.606	0.014	0.170	5 vs 5	0.760	0.016	0.131

The receiver operating characteristics (ROC) area under the curve (AUC) are reported for each evaluation

Four microRNAs were associated with AD, but mir-191-3p was also associated with NSCLC metastatic. Seven were associated with NSCLC, where five were unique for contrasts with NSCLC (mir-103a-3p, mir-191-5p, mir-185-5p, mir-20a-5p and miR-144-5p). Three were associated with SCLC, where one, miR-487a-3p was downregulated in SCLC versus all controls (Table 2). This miR was downregulated with zero expression in 95% and 92% SCLC cases and 74.8% and 61.5% controls in the discovery and validation serum datasets, respectively (Supplementary Excel file 3 and 4).

### Combining biomarkers alone and with clinical variables

An evaluation of the lung cancer predictive performance of the 15 SDE microRNAs combined (Table 2) was performed on the complete discovery and validation datasets, and AUC of 0.708 (95% CI 0.643–0.773) and 0.703 (95% CI 0.582–0.821) were achieved, respectively (Table 3). When these 15 microRNAs were combined with the original eight clinical variables of the HUNT Lung Cancer Model (sex, age, body-mass index (BMI), pack-years, number of cigarettes per day, quit time in years, hours of daily indoors smoke exposure and history of daily cough in periods through the year), (Markaki et al. 2018) the AUC increased to 0.722 (95% CI 0.656–0.785) in the discovery dataset, but decreased in the validation serum dataset with an AUC of 0.697 (95% CI 0.573–0.812) compared to the 15-microRNA signatures (Table 3).

**Table 3:** Lung cancer risk prediction performance in the discovery (N = 238) and the validation (N = 72) serum datasets by the 15-microRNA signature, and the combination of the 15 microRNAs and the original eight clinical variables in the HUNT Lung Cancer Model (sex, age, body-mass index (BMI), pack-years, number of cigarettes per day, quit time in years, hours of daily indoors smoke exposure and history of daily cough in periods through the year)

Contrast	SDE miRNA	Clinical variables	Combined microRNA		Combined microRNAs + clinical variables	
			Discovery serum dataset N = 238 Cases N = 119 Controls N = 119	Validation serum dataset N = 72 Cases N = 36 Controls N = 36	Discovery serum dataset N = 238 Cases N = 119 Controls N = 119	Validation serum dataset N = 72 Cases N = 36 Controls N = 36
			AUC (CI 95%)	AUC (CI 95%)	AUC (CI 95%)	AUC (CI 95%)
	miR-103a-3p	Age, gender, pack-years, daily cough parts of the year, hours of indoor smoke exposure, quit time in years, number of cigarettes daily, body mass index (BMI)	0.708 (0.643–0.773)	0.703 (0.582–0.821)	0.722 (0.656–0.785)	0.697 (0.573–0.812)
All cases vs all controls	miR-1228-5p					
	miR-1306-5p					
	miR-144-5p					
	miR-152-3p					
	miR-185-5p					
	miR-191-3p					
	miR-191-5p					
	miR-20a-5p					
	miR-4485-3p					
	miR-487a-3p					
	miR-511-5p					
	miR-598-3p					
	miR-616-3p					
	miR-8072					

The receiver operating characteristics (ROC) area under the curve (AUC) are reported for each evaluation

**Fig. 2:**
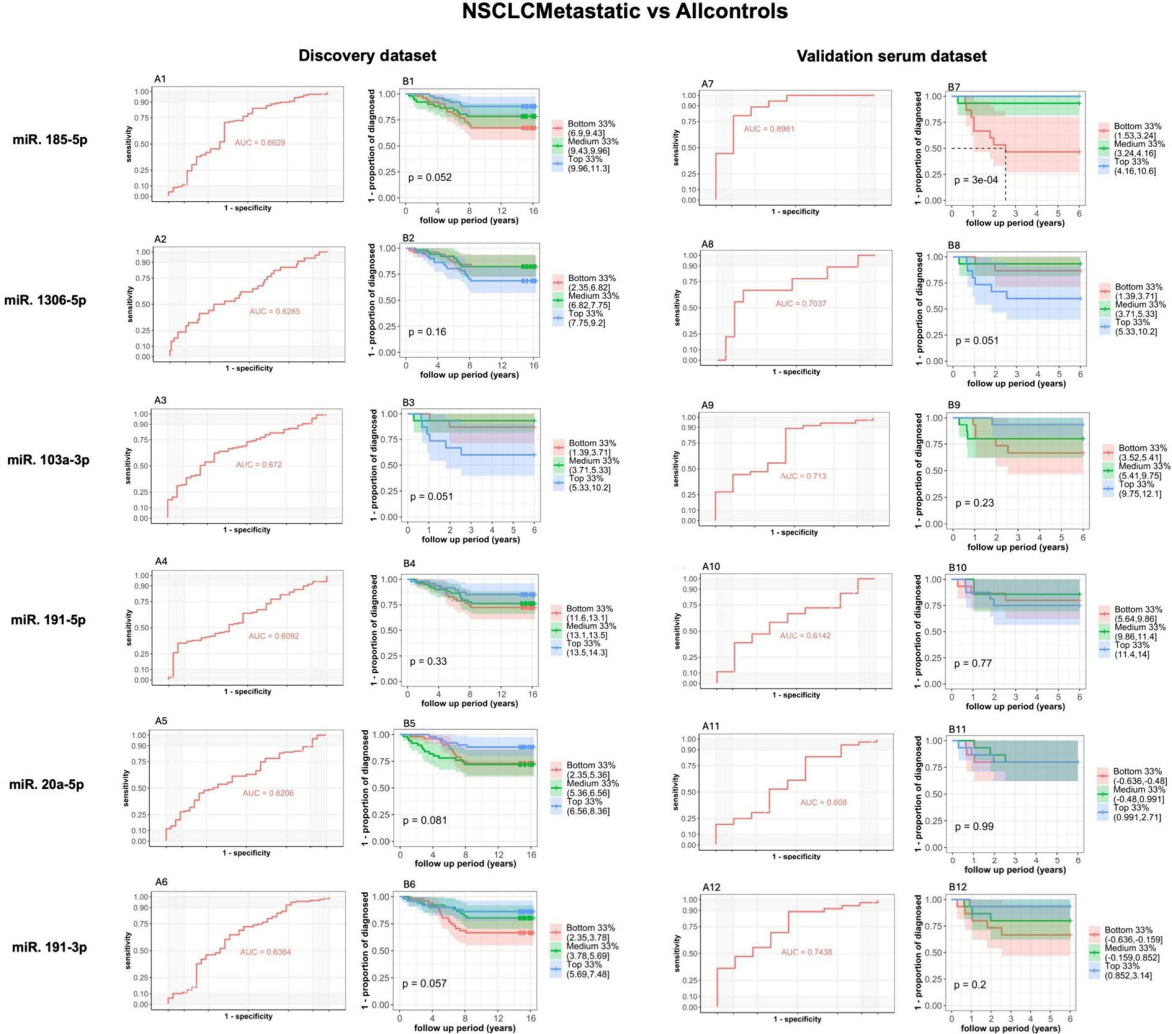
Significantly differentially expressed (SDE) microRNAs in the contrast NSCLCMetastatic vs all controls in the discovery and the validation serum datasets. A1–12: AUC values. B1–12: Kaplan Meier curves indicating the proportionality of diagnosis among individuals grouped by their raw expression in log2 scale in three quantiles; low, medium and high

The contrast “NSCLC metastatic versus all controls” had a total of six SDE microRNAs, the miR-103a-3p, miR-1306-5p, miR-185-5p, miR-191-3p, miR-191-5p and miR-20a-5p which independently showed an AUC > 0.6 in both the discovery and in the validation serum datasets (Fig. 2).

Combined, the 6-microRNA signature yielded an AUC of 0.777 (95% CI 0.675–0.868) and 0.806 (95% CI 0.654–0.932) (Table 4) in the discovery and validation serum datasets, respectively. Furthermore, when these six microRNAs were combined with the original eight clinical variables of the HUNT Lung Cancer Model (Markaki et al. 2018), the AUC increased to 0.79 (95% CI 0.694–0.876) and 0.833 (95% CI 0.698–0.948) in the discovery and validation serum datasets, respectively (Table 4).

**Table 4:** Metastatic non-small cell lung cancer risk prediction performance in the discovery (N = 198) and the validation (N = 60) serum datasets by the combination of the six significantly differentially expressed (SDE) microRNAs in the contrast “NSCLCMetastatic vs all controls” and the original eight clinical variables in the HUNT Lung Cancer Model (sex, age, body-mass index (BMI), pack-years, number of cigarettes per day, quit time in years, hours of daily indoors smoke exposure and history of daily cough in periods through the year)

Contrast	SDE miRNA	Clinical variables	Combined microRNA		Combined microRNAs + clinical variables	
			Discovery serum dataset N = 198 Cases N = 79 Controls N = 119	Validation serum dataset N = 60 Cases N = 24 Controls N = 36	Discovery serum dataset N = 198 Cases N = 79 Controls N = 119	Validation serum dataset N = 60 Cases N = 24 Controls N = 36
			AUC (CI 95%)	AUC (CI 95%)	AUC (CI 95%)	AUC (CI 95%)
NSCLCMetastatic vs all controls	miR-103a-3p	Age, gender, pack-years, daily cough parts of the year, hours of indoor smoke exposure, quit time in years, number of cigarettes daily, body mass index (BMI)	0.777 (0.675–0.868)	0.806 (0.654–0.932)	0.790 (0.694–0.876)	0.833 (0.698–0.948)
	miR-1306-5p					
	miR-185-5p					
	miR-191-3p					
	miR-191-5p					
	miR-20a-5p					

The receiver operating characteristics (ROC) area under the curve (AUC) are reported for each evaluation

### Pre-diagnostic plasma validation datasets in NOWAC and NSHDS

The analysis in pre-diagnostic plasma datasets did not validate the serum analyses. The microRNAs were tested in the two plasma sample sets one by one, and none reached significance. Moreover, most had fold-change in the opposite direction from the serum samples but retained the direction between the two plasma sample sets (Supplementary File 5 and 6). We concluded that there are probably biological differences between plasma and serum samples, and therefore they cannot be used for validating the serum results.

## Discussion

Lung cancer should be diagnosed early to increase chances of survival. Here we present a group of fifteen newly discovered microRNAs in pre-diagnostic serum of current and former smokers in HUNT, that were validated in a separate cohort. This may be developed to facilitate early diagnosis of lung cancer. Plasma is not suitable as these candidate miRNAs were not validated in pre-diagnostic plasma specimens from the NOWAC and NSHDS studies.

The design of the study included selection of cases and controls among ever-smokers only, because they comprise more than 90% of lung cancer cases in Norway, according to large population-based studies (Markaki et al. 2018). All cases and controls were ever-smokers with an estimated high risk for lung cancer and matched for several variables. By using our HUNT Lung Cancer Model risk calculator, the median risk for developing lung cancer in cases and controls was not significantly different, 1.6% and 1.25% in six years, respectively (p = 0.795, Table 1). Therefore, the challenge to find relevant biomarkers in this study may be harder than using e.g. never-smokers in the control group. However, the chance to find true biomarkers, not confounded by smoking or other clinical factors is then also higher.

By means of miRseq of pre-diagnostic serum, we identified a multitude of SDE microRNAs in non-symptomatic, apparent healthy persons 0–8 years before diagnosis. Fifteen microRNAs with AUC > 0.60 were validated in an independent, similar serum dataset with shorter time to diagnosis, 0–3.8 years. We found that the average AUC values among common SDE microRNAs in the same direction in the various contrasts was higher in the validation serum dataset with shorter time to diagnosis compared to in the discovery dataset. This could indicate that the biomarker´s predictive value increases as one approaches the clinical diagnosis.

### Significant differentially expressed (SDE) microRNAs and histological subtypes

The selected case control groups were balanced, and represented the largest histological subtypes, AD, SQ and SCLC, as we hypothesised that each subtype would have a different serum profile. Each case had a matched control, so that even small case–control groups would be informative. These requirements were followed in both study serum datasets. There were significant microRNAs in contrast involving all subtypes (Supplementary File 3). The analysis against all controls is the most similar to a real-life biomarker situation and therefore we chose to focus on the microRNAs that were significant against all controls. Among these, most SDE validated microRNAs were found among the AD, NSCLC and SCLC subtypes, and only one single was found in the SQ subtype. Interestingly, most validated SDE were downregulated in the subject with pre-lung cancer condition (Table 2), which fits with the notion that microRNAs are predominantly downregulated in tumors (Williams et al. 2017).

One SDE validated microRNAs, miR-4485-3p, was found among the SQ cases. Interestingly the SQ cases were predominantly diagnosed at an early stage (60% vs 28%, Supplementary Table 3), proposing that miR-4485-3p may serve as a diagnostic biomarker of early stage SQ. However, to our best knowledge, this association has not been reported before, and therefore needs further validation. The AD has a more heterogeneous biology, often more aggressive and grows more peripheral, with less symptoms in early stages. Here, most cancers were diagnosed later (26% early vs 59% late). There were six SDE in NSCLC metastatic and one in all cases metastatic, which may indicate that the late stage disease, independently of tumor subtype, introduces differential expression of microRNAs. Finally, SCLC has two upregulated microRNAs in the non-metastatic stage and one in the total SCLC group. In SCLC there were also more late-stage tumors diagnosed (25% early vs 53% late). SCLC is the most aggressive type, often growing within weeks or a few months to advanced disease, and in yearly CT screening programs this is the typical interval cancer (Aberle et al. 2011; de Koning et al. 2020; Silva et al. 2016). Regarding time to diagnosis, the goal was to discover biomarkers that could diagnose, predict or prognose lung cancer of any major subtype some years prior to the clinical diagnosis. This poses a challenge as a small, asymptomatic tumor may not induce significant changes in microRNA profile in the serum as an advanced metastatic tumor.

### MicroRNA signatures as potential biomarkers

Several groups have reported different microRNA signatures as potential biomarkers for early diagnosis of lung cancer (Halvorsen et al. 2016; Pan et al. 2018) where these panels achieved higher sensitivity and specificity compared to single microRNAs (Han and Li 2018). This is consistent with our findings, where a signature of 15 and six microRNAs increased the predictive performance of lung cancer and metastatic NSCLC, respectively, compared to the single microRNAs except one. Furthermore, by combining both the 15- and 6-microRNA signatures with the original eight clinical variables of the lung risk prediction model, HUNT Lung Cancer Model, the lung cancer predictive performance further increased to AUC > 0.70, suggesting that microRNAs in combination with clinical variables potentially can improve lung cancer risk prediction. This is supported by the recent results from Yu et al. (2022) which showed that microRNAs can have an independent risk stratification beyond clinical information such as age, smoking history, family history of lung cancer and other variables used in lung cancer risk prediction models. They reported that a signature of three microRNAs (miR-142-3p, miR-148a-3p and miR-451a) could substantially improve lung cancer risk prediction of eight different lung cancer risk prediction models (LLPi, Pittsburg Predictor, Bach, PLCOm2012, LLP, Hoggart, Spitz, LCRAT), with an AUC improvement between 0.041 to 0.096 where the highest optimism corrected AUC was of 0.762 for the combination miR-score + LPP, Pittsburg Predictor or LCRAT (Yu et al. 2022). However, further studies are needed to verify whether the microRNA signatures in the present study can improve the lung cancer predictive performance of the HUNT Lung Cancer Model.

### Differences in microRNA expression and levels between serum and plasma samples

A recent study by Wakabayashi et al. analysing total microRNAs showed significant differences in serum and plasma levels for around one third of the microRNAs tested (Wakabayashi et al. 2024). Furthermore, they observed significant time-dependent changes of microRNA levels in plasma and not in serum; about 20% of the microRNAs tested tended to decrease in plasma with time during the 3 h period after blood collection (Wakabayashi et al. 2024). These differences in serum and plasma levels of microRNAs might be due to inherent biological differences as well as in differences in sample processing and analysis. This may explain why we could not validate our findings in plasma samples from the NOWAC and NSHDS studies. However, we cannot rule out the possibility that other studies may have different findings.

### Comparison with published signatures

Up to date several studies have found diagnostic microRNAs for NSCLC, AD and SQ, using serum or plasma at diagnosis (Bottani et al. 2019; Zhong et al. 2021). However, there are few overlapping findings between these studies (Bottani et al. 2019; Zhong et al. 2021). A recent large study of a 5-microRNA signature that was validated in a Chinese and Caucasian population did not overlap with other similar studies, and not with our study (Ying et al. 2020). A validated 24-microRNA miR-Test, designed to discern between benign and pathological nodules detected by lung cancer screening (Sozzi et al. 2014b), had no overlap with the 15 microRNAs we found. This may be due to different microRNA expression years before clinical cancer rather than at diagnosis. It is known that pre-diagnostic microRNAs are highly dynamic in lung cancer patients and can be histology and stage dependent (Umu et al. 2020). There might also be due to the already mentioned difference of microRNA levels between serum and plasma samples, as well as issues regarding the time period and platform used for sequencing between studies as we know that the technology has evolved rapidly. Batch effects are common in microRNA sequencing that may alter outcomes between studies (Johnson et al. 2006).

### MicroRNA in current versus former smokers, males versus females and age differences.

Smoking can affect the microRNA expression (Wu et al. 2019). Several studies have reported very good results on discerning between cases and controls, but many of them have not taken into account the important confounding effect of smoking status (Wozniak et al. 2015; Ying et al. 2020). Thus, some of the published signatures may reflect smoking status rather than cancer. In our study we corrected for current versus former smoking to avoid potentially false discoveries. Studies have also shown strong correlation of some microRNAs with both gender and age (Rounge et al. 2018), therefore we corrected for those two factors as well.

### Impact of sample processing and storage

The serum samples used in our study have been stored in liquid nitrogen or − 80 °C freezers 20–22 years before analysis with two freeze and thaw cycles. A study on microRNAs in serum stored in ultra-low temperatures for up to 17 years showed no statistically significant changes of most microRNAs (Matias-Garcia et al. 2020). Moreover they found that miR-451a levels were altered due to contamination during sampling and that freeze-thawing of one to four cycles showed an effect only on miR-30c-5p. None of these microRNAs were significant in our study. Also, there are no large differences in storage time between samples. Thus, storage does not seem to introduce any significant bias in our study. Hemolysis is regarded as a source of bias in serum microRNA analysis as some microRNAs are abundant in red blood cells (Kirschner et al. 2013; Pizzamiglio et al. 2017), these include mir-16, miR-21, mir-17, mir-92a, mir-106a, mir-320, mir-324-3p, mir-451 and mir-486 (Kirschner et al. 2013; Pizzamiglio et al. 2017). The miR-320 has also been found overexpressed in serum of smokers versus never smokers (Suzuki et al. 2016). None of the significant microRNAs were found among these.

### Strengths

There are several published papers on using circulating microRNAs for early diagnosis of lung cancer (Bianchi et al. 2011; Seijo et al. 2019; Wang et al. 2015). However, lack of follow-up studies and validations have hampered their clinical implementation. Moreover, true pre-diagnostic samples collected under standard conditions and comprehensive clinical variables are scarce. Here, we have the advantage of the population-based prospective HUNT study that included the majority of the adult population in one Norwegian county in several waves with ten-year intervals and a follow-up time of up to 16 years. Importantly, the serum collected was accompanied by vital data and almost 200 questions on health and lifestyle were answered by each participant. In the HUNT2 wave (1995–1997) and HUNT3 wave (2005–2008), more than 65,000 and 33,000 unique participants were included, respectively.

In a screening program, one of the worries is that one discovers indolent lung cancers that may not be lethal. In this study all cases had been diagnosed by clinical presentation as there is no screening program in Norway. Therefore, the microRNAs discovered here are linked to lethal cancers (Supplementary Fig. 2). Importantly, we also had detailed smoking history on all participants and therefore could correct for smoking status, which is important for serum microRNA expression (Wu et al. 2019). Likewise, age and sex-specific microRNAs have been shown (Rounge et al. 2018), and therefore it was very important to correct for this. We also combined multiple SDE microRNA and clinical variables that are included in a validated lung cancer risk prediction model, the HUNT Lung Cancer Model (Markaki et al. 2018).

It is also important to point out that the material in our study consist of serum samples from both individuals that subsequently were diagnosed with metastatic as well as non-metastatic lung cancer. This reflects the real world setting where we have lung cancer cases with different biology and natural courses, including cases with rapid development as well as cases with longer interval from debut/localized to metastatic disease.

### Limitations

The main limitation of this study is the relative low number of participants in the validation set. This may be the reason for not finding more overlapping significant microRNAs in this dataset. The scarcity of pre-diagnostic samples a short time before diagnosis is the main reason for the small number of participants. However, contrary to several other studies that use few samples for high-throughput discovery, we had a quite large and well-defined population for discovery, thus lowering the risk for false positives and false negative findings, being able to validate 15 microRNAs in the validation dataset. The purpose of early diagnosis of lung cancer is to detect tumors before they metastasize. It would therefore be of great interest to test the performance of the microRNA signatures between controls and future non-metastatic (stage I and II) cases. Subdividing the validation serum dataset samples according to stage would lead to quite small sub-cohorts (Supplementary Table 3), making it very challenging to draw statistically valid conclusions. Thus, the limited size of the validation serum dataset left this untested. However, the samples used in the study are all pre-diagnostic samples collected up to several years before clinical diagnosis. In the discovery serum dataset, the expected time to diagnosis was 4.87, 5.69, and 4.095 years for AD, SCLC, and SQ cases, respectively (see Supplementary), therefore the microRNA identified in the pre-diagnostic serum samples are most likely present before metastatic disease. Consequently, the microRNA candidates could represent biomarkers for early stage diagnosis as well. A limitation regarding the microRNA expression is that some significant miRNAs had very low counts. However, the results should be robust, as all validated microRNAs retained their significance after correction for library size. The FDR significance threshold was set to 0.25 in this study. While a 0.25 threshold represents a broad, liberal search that may include many false positives, requiring that findings must be confirmed in the validation datasets with the same deregulation direction should largely discard many false positives.

### In conclusion

This study revealed novel serum biomarkers for AD, SQ, SCLC and NSCLC which were validated in pre-diagnostic serum samples up to 3.8 years before clinical diagnosis of lung cancer in HUNT. They were not validated in plasma in other pre-diagnostic samples and thus plasma is not suitable for this analysis. The lung cancer predictiveness of the microRNAs increased by combining multiple microRNAs to a signature and combining with the original eight clinical variables from the HUNT Lung Cancer Model. To be able to go forward with some of these biomarkers for early clinical diagnosis, they need to be further validated in larger pre-diagnostic serum datasets before clinical implementation can be considered.

### Supplementary Information

Below is the link to the electronic supplementary material.

Supplementary file1 (XLSX 10 KB)

Supplementary file2 (XLSX 145 KB)

Supplementary file3 (PNG 17 KB)

Supplementary file4 (PNG 10 KB)

Supplementary file5 (XLSX 10 KB)

Supplementary file6 (XLSX 10 KB)

Supplementary file7 (DOCX 1621 KB)

## Data Availability

In agreement with the license agreements applicable to this study, only the named authors were given full access to the data during the study. This is to ensure that all personal and health information of the participants in the HUNT2, HUNT3, NOWAK and NSHDS is kept confidential. The raw data from the HUNT2, HUNT3, NOWAK and NSHDS cohorts could be accessed upon reasonable request to the originating cohorts. Access will be subject to compliance with local ethical and security policies.
