# Supplementary figures and images for: Promising microRNAs in pre-diagnostic serum associated with lung cancer up to eight years before diagnosis: a HUNT study
Source: J Cancer Res Clin Oncol. 2024 Jul 20;150(7):355. doi: 10.1007/s00432-024-05882-4 (PMC11271336; doi:10.1007/s00432-024-05882-4)

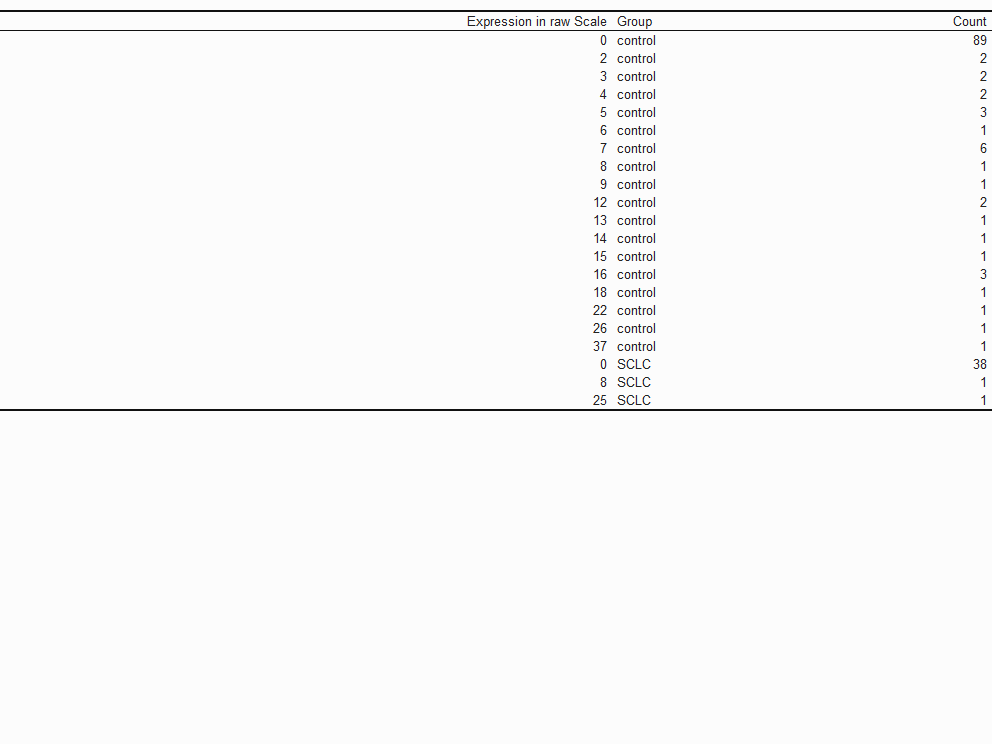

Supplement: Supplementary file 3 — Supplementary file3 (PNG 17 KB) [file 432_2024_5882_MOESM3_ESM.png]

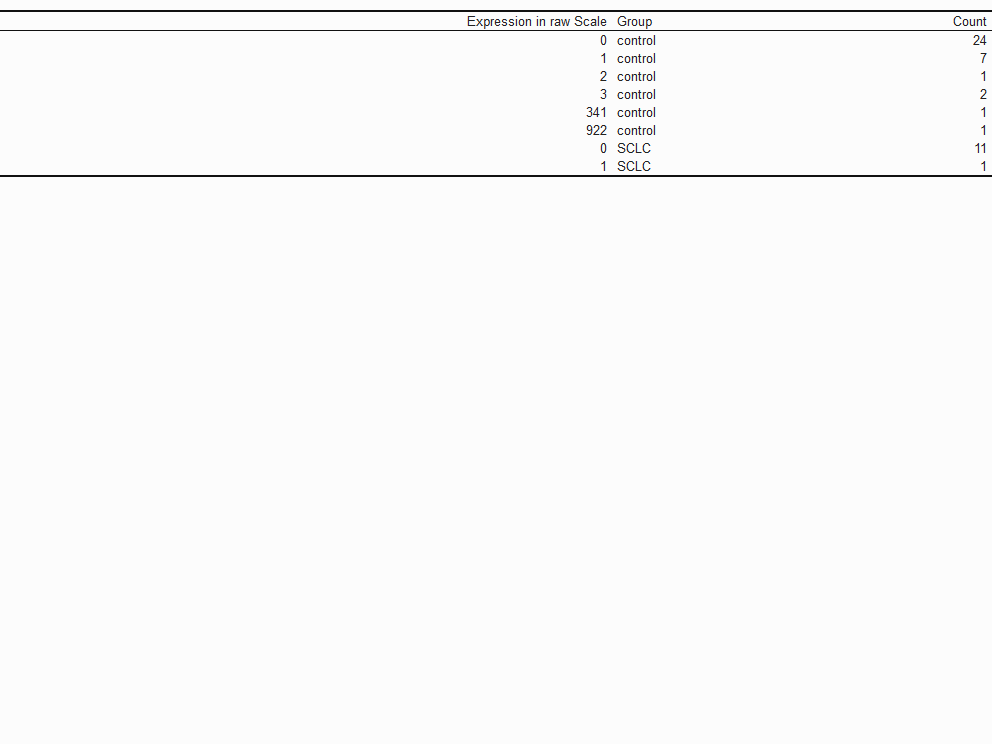

Supplement: Supplementary file 4 — Supplementary file4 (PNG 10 KB) [file 432_2024_5882_MOESM4_ESM.png]
