# Supplementary material for: Promising microRNAs in pre-diagnostic serum associated with lung cancer up to eight years before diagnosis: a HUNT study
Source: J Cancer Res Clin Oncol. 2024 Jul 20;150(7):355. doi: 10.1007/s00432-024-05882-4 (PMC11271336; doi:10.1007/s00432-024-05882-4)
Supplement: Supplementary file 7 — Supplementary file7 (DOCX 1621 KB) [file 432_2024_5882_MOESM7_ESM.docx]

**Promising microRNAs in pre-diagnostic serum associated with lung cancer up to eight years before diagnosis: A HUNT study.**

**Ioannis Fotopoulos, Olav Toai Duc Nguyen, Therese Haugdahl Nøst, Maria Markaki, Vincenzo Lagani, Robin Mjelle, Torkjell Sandanger, Pål Sætrom, Ioannis Tsamardinos, Oluf Dimitri Røe**

**Supplementary Materials**

**Supplementary Methods**

**Differential Expression Analysis**

We employed a widely known RNA-seq analysis pipeline (Law et al. 2016) to perform univariate differential expression analysis. At its core, the pipeline utilizes a moderated t-test and linear models to identify deregulated quantities, adjusting for the variations of exogenous factors (e.g., batch effects), and over-under expressions due to technically biological laboratory effects. The pipeline is constituted of five successive steps (Supplementary Schema 1): (i) filtering of non-expressed microRNAs, (ii) within-sample normalisation (Robinson and Oshlack 2010) (iii) estimation of gene-specific mean-variance correlations (Law et al. 2014; Liu et al. 2015), (iv) linear modelling with Bayesian modelling of gene-wise variations (Ritchie et al. 2015), (v) multiple hypotheses testing correction.

We consider a microRNA as statistically differentially expressed (SDE) in discovery cohorts at 0.25 FDR level. The differential expression analysis results obtained in the discovery dataset (HUNT2, N=238) are validated in an external validation dataset (HUNT3, N=72).


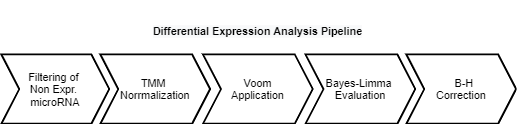


**Supplementary Schema 1.** The five-step statistical modeling pipeline is considered for the current microRNA statistical analysis.

*Filtering out lowly and non-expressed microRNAs:* we require each microRNA to have 1 read count in at least 39 samples out of 238 in the discovery cohort and in at least 12 samples out 72 in the big validation Cohort (I).

*Within sample normalisation:* we apply the TMM method as presented in it original publication (Robinson et al. 2010; Robinson and Oshlack 2010).

*Estimation of gene-specific mean-variance correlations:* the Voom framework (Liu et al. 2015) addresses the heteroscedasticity between the variance and mean expression present in (micro)RNA-seq data, accounting also for sample variabilities, by estimating gene and sample specific weights that are used in the subsequent linear modelling step.

*Linear modelling*: the limma package (Ritchie et al. 2015) models log-transformed expressions values according to the experimental design and accounting for the voom sample and gene specific weights. Bayesian inference is further used for robustly estimate gene variances.

Multiple testing correction: all p-values were adjusted with the Hochberg-Bonferroni procedure (Benjamini and Hochberg 1995).

**Validation Procedure**

For each contrast, we aim at identifying statistically significant differentially expressed (DE) microRNA in the discovery cohort. Furthermore, we want to validate them in the external independent validation cohort. First, we identify DE microRNAs in the discovery cohort with false discovery rate (FDR) <0.25. Out of these groups, we keep only the DE microRNAs which have identical log fold change (logFC) sign in both discovery and validation cohort. We define this set of N microRNAs as candidate biomarkers. These candidate biomarkers are further filtered as follows:

- AUC on discovery cohort >0.6

- Restricted FDR on validation cohort <0.25. “Restricted” means that the FDR is computed only over the N p-values

of the candidate biomarkers

Only the candidate biomarkers that satisfy all these conditions are considered validated.

**Supplementary Results**

**Staging Information**

| Code | Definition | Stage definition |
| --- | --- | --- |
| 0 | No invasion to the neighboring tissues or organs, no lymph nodes or organ metastasis.  Metastasis in the same organ as the origin of the primary tumor. | Non-metastatic  Stage I-IIB |
| 1 | Lymph node metastasis in the same body section as the primary tumor. |  |
| 2 | Lymph node metastasis to a different body section than the primary tumor. | Metastatic  Stage III-IV |
| 3 | Organ metastasis in the same body section as the primary tumor |  |
| 4 | Organ metastasis to a different body section than the primary tumor. |  |

**Supplementary Table 1.** Metastasis codes of the Norwegian Cancer Registry with general definitions used from 01.01.1986. Code 0-1 is a surrogate for Stage I-IIB and Code 2-4 for Stage III-IV. Code 0 also may include few cases with metastasis in the same organ, but no other metastases.

**Descriptive Statistics on Clinical Data**

We examine if there are any distributional differences in the clinical risk factors related to lung cancer development between discovery and validation cohorts (see Supplementary Fig. 1). According to Markaki et al. (2018) the clinical variables that are significantly associated with lung cancer development are gender, age, smoking habits (pack-years, smoking cut years, smoking status), and body mass index (BMI). The clinical variables that gauge the hours of exposure in a smoky room (SmoExpH) and the indication of cough daily (CougDy) on yearly basis have not been included in the descriptive analysis due to their absence in the validation cohort. Therefore, we conducted the Tukey’s and chi-square statistical tests for the continuous and binomial variables respectively.

The results showed statistically significant heterogeneity in 3 out of the 6 examined variables across the discovery and validation cohorts at 0.05 significance level (Supplementary Table 2). Particularly, we observe that age is significantly different between cohorts, with the participants in the discovery dataset being five years older on average. There is also an unbalanced distribution in terms of gender, with 64.7% and 44.4% men in the discovery and validation cohort populations respectively.

| **Multiple pairwise Tukey test** | | | | |
| --- | --- | --- | --- | --- |
| **Continuous trait** | **p-value** | | **95% CI** | **statistic** |
| PartAg | 0.000213 | | [2.17 - 8.167] | 5.169 |
| SmoPackYrs | 0.101739 | | [-0.42 -8.94] | 4.259 |
| Smoking Cessation Years (SmoDyCesDu) | 0.047808 | | [-0.362 - 4.816] | 2.22 |
| BMI | 0.336068 | | [-1.594 - 0.546] | -0.524 |
| **Chi-square statistical test** | | | | |
| **Nominal trait** | | **p-value** | **statistic** |  |
| Sex | | 0.0033 | 8.629 |  |
| Smoking Exposure at Childhood | | 0.835 | 0.0431 |  |

**Supplementary Table 2.** Tukey’s and chi-square statistical tests for the continuous and binomial variables in discovery and validation dataset. P<0.05 for statistically significant differences among variables.


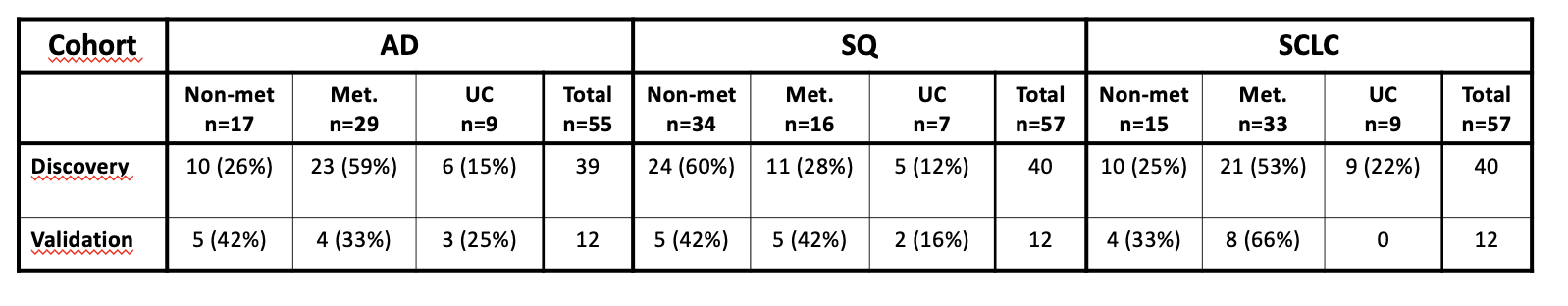


**Supplementary Table 3.** Number of non-metastatic (“Non-met”; Stage I-IIB) and metastatic (“Met.”; Stage III-IV) cases across the different types of lung cancer under examination. Some cases were unclassifiable according to stage.

UC =Unclassifiable cases.


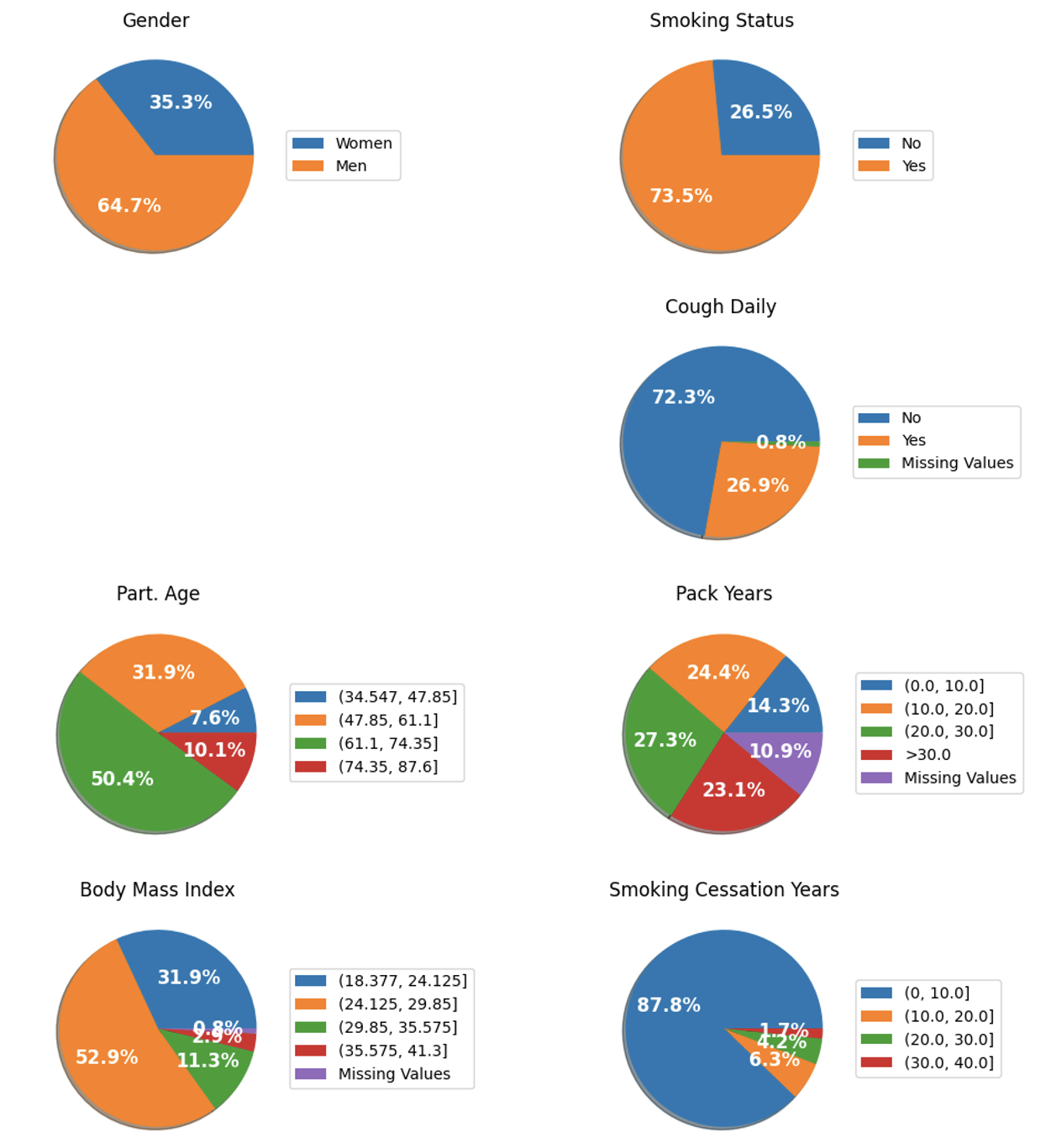


**Supplementary Fig. 1** Descriptive statistics, per clinical factor, obtained from the discovery cohort. The smoking status refers to current (Yes) and former (No) smokers.

**Time to diagnosis**

No differences in terms of survival times (0.72 p-value, log rank test) are identified amongst lung cancer subtypes (AD, SCLC, SQ) in the discover cohort. Expected time to diagnosis was 4.87, 5.69, and 4.095 years for AD, SCLC, and SQ cases respectively (Supplementary Figure 2). Additionally, no indication of statistically significant differences was observed between the Kaplan Meier estimates in the validation cohort (0.59 p-value, log rank test) (Supplementary Figure 2), where the expected time to diagnosis was 1.01, 1.901, 1.16 years for AD, SQ, and SCLC subtypes.

*
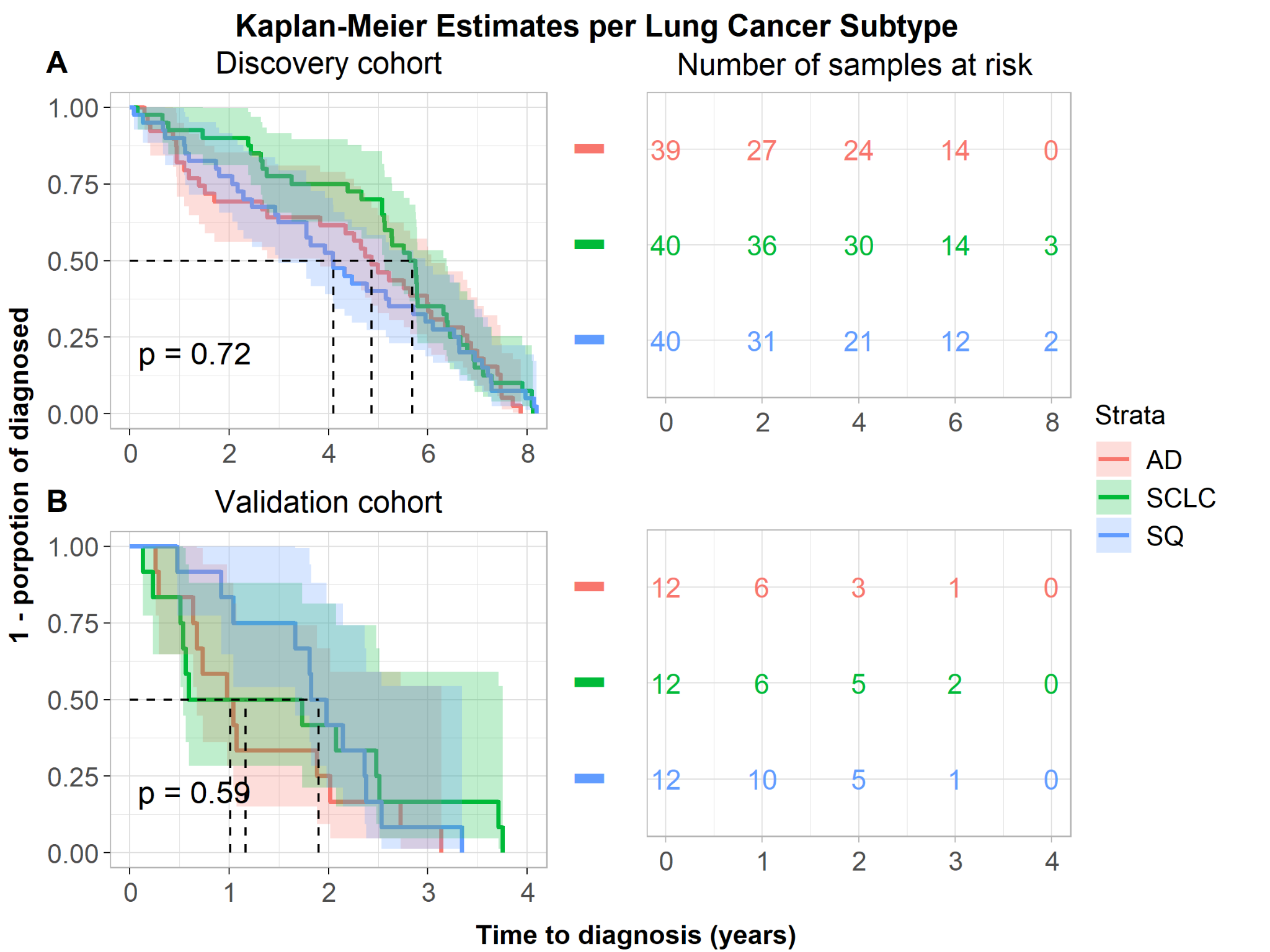
*

**Supplementary Fig. 2** Time to event (lung cancer diagnosis) in both discovery and validation cohort according to adenocarcinoma (AD), squamous cell carcinoma (SQ), and small cell lung cancer (SCLC) subtypes.

X-axis: years from serum sampling (time zero).

Y-axis: Proportion of undiagnosed (1 - proportion of diagnosed).

A. Discovery cohort. B. Validation cohort.

**Descriptive Statistics on Molecular Data**

**
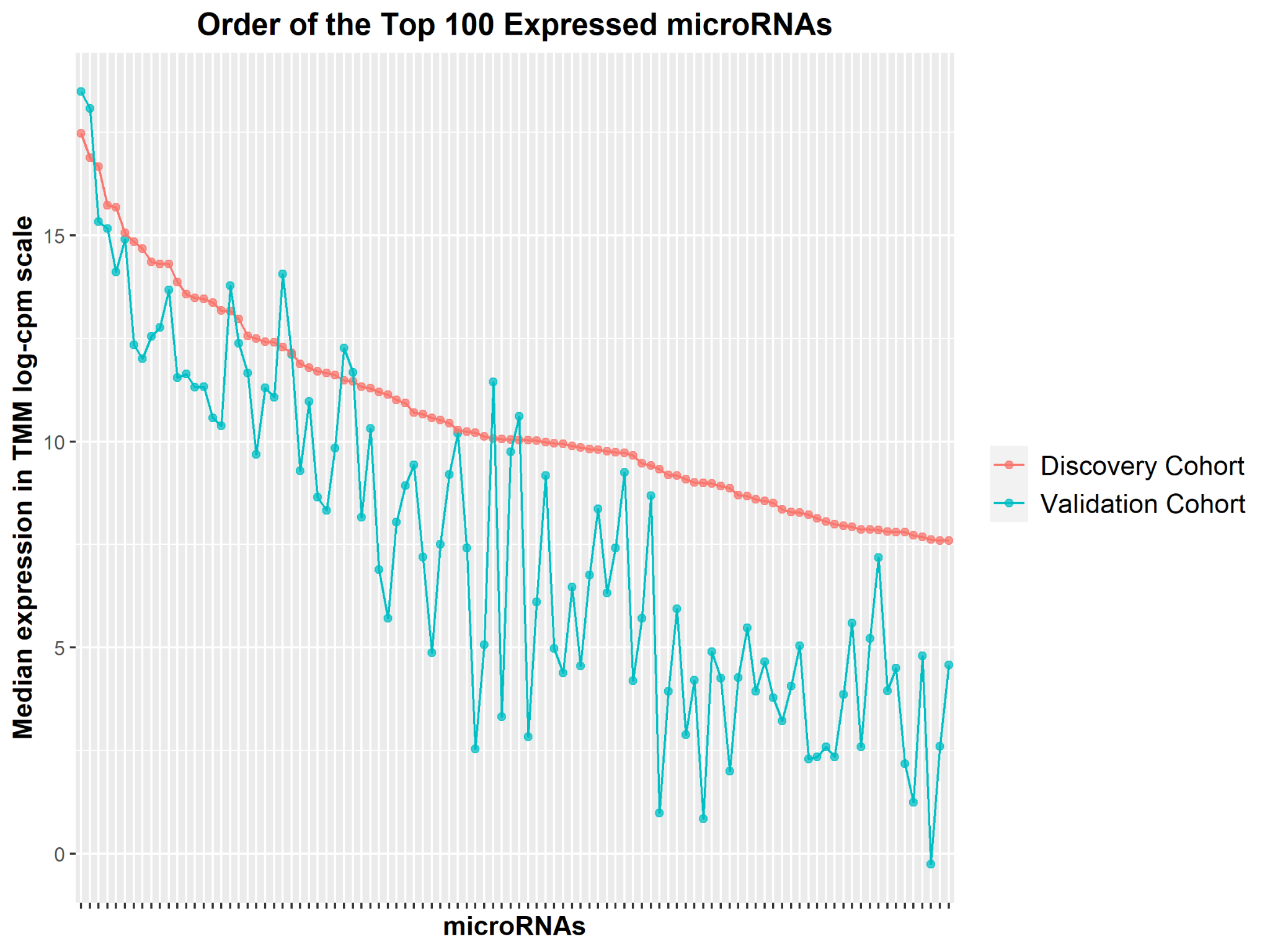
**

**Supplementary Fig. 3** The order of the normalized [2] log-cpm expression of the top 100 expressed microRNA in the discovery dataset (n = 238), and the corresponding expression of the common microRNAs in the validation dataset (n = 72). We observe visually a downward trend in the expressions amongst the microRNAs in the validation cohorts.


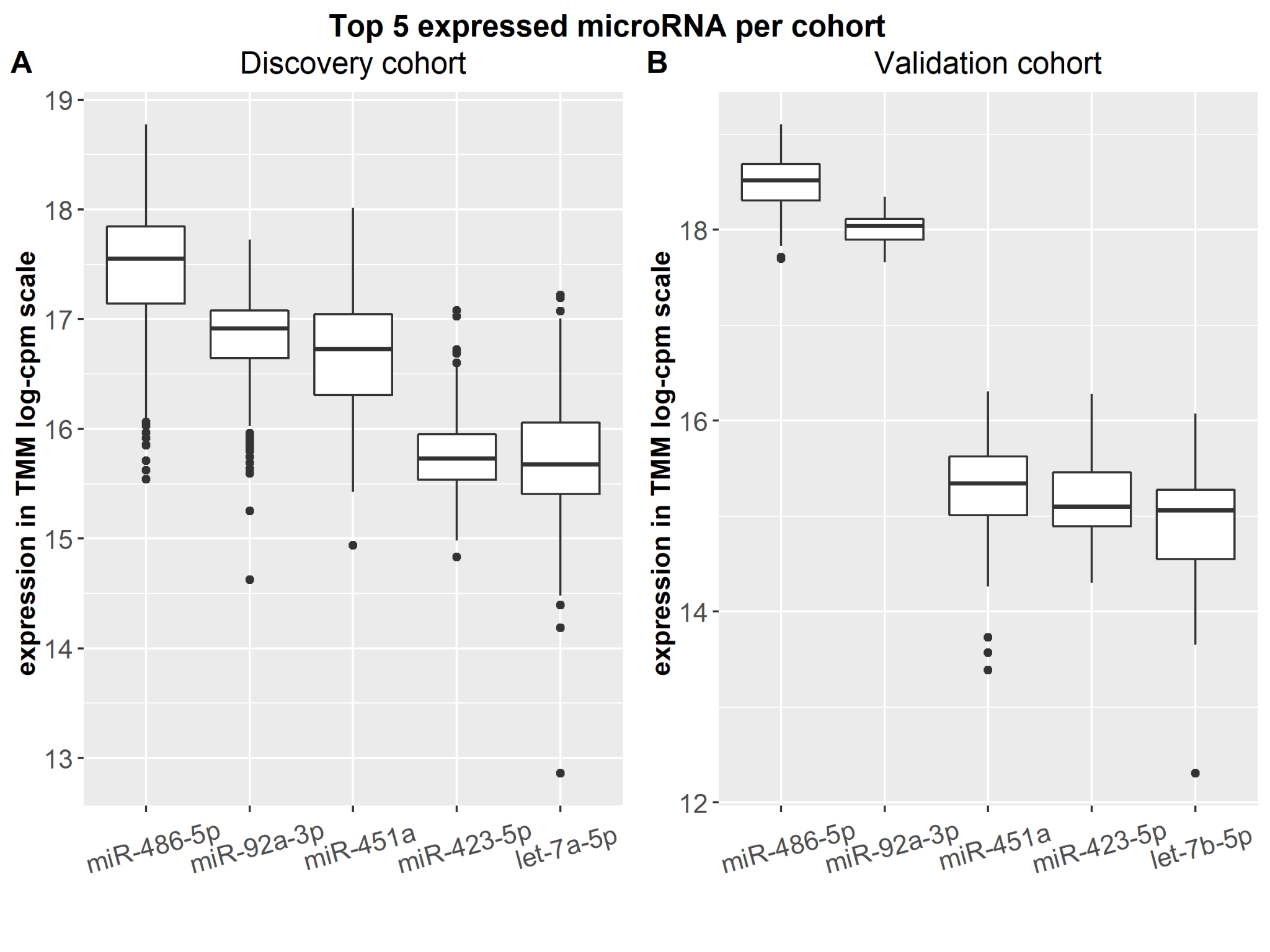


**Supplementary Fig. 4** Top 5 expressed microRNA in the discovery and validation dataset. The order is based on the median expression.

**References**

Benjamini Y, Hochberg Y. Controlling the False Discovery Rate: A Practical and Powerful Approach to Multiple Testing. Journal of the Royal Statistical Society: Series B (Methodological). 1995;57:289-300. https://doi.org/https://doi.org/10.1111/j.2517-6161.1995.tb02031.x

Law CW, Alhamdoosh M, Su S, Dong X, Tian L, Smyth GK, et al. RNA-seq analysis is easy as 1-2-3 with limma, Glimma and edgeR. F1000Res. 2016;5. https://doi.org/10.12688/f1000research.9005.3

Law CW, Chen Y, Shi W, Smyth GK. voom: Precision weights unlock linear model analysis tools for RNA-seq read counts. Genome Biol. 2014;15:R29. https://doi.org/10.1186/gb-2014-15-2-r29

Liu R, Holik AZ, Su S, Jansz N, Chen K, Leong HS, et al. Why weight? Modelling sample and observational level variability improves power in RNA-seq analyses. Nucleic acids research. 2015;43:e97. https://doi.org/10.1093/nar/gkv412

Markaki M, Tsamardinos I, Langhammer A, Lagani V, Hveem K, Roe OD. A Validated Clinical Risk Prediction Model for Lung Cancer in Smokers of All Ages and Exposure Types: A HUNT Study. EBioMedicine. 2018;31:36-46. https://doi.org/10.1016/j.ebiom.2018.03.027

Ritchie ME, Phipson B, Wu D, Hu Y, Law CW, Shi W, et al. limma powers differential expression analyses for RNA-sequencing and microarray studies. Nucleic acids research. 2015;43:e47. https://doi.org/10.1093/nar/gkv007

Robinson MD, McCarthy DJ, Smyth GK. edgeR: a Bioconductor package for differential expression analysis of digital gene expression data. Bioinformatics. 2010;26:139-40. https://doi.org/10.1093/bioinformatics/btp616

Robinson MD, Oshlack A. A scaling normalization method for differential expression analysis of RNA-seq data. Genome Biol. 2010;11:R25. https://doi.org/10.1186/gb-2010-11-3-r25
